# Neuroprotective Effects of Zinc Oxide Nanoparticles in a Rotenone-Induced Mouse Model of Parkinson's Disease

**DOI:** 10.7150/ntno.95863

**Published:** 2024-06-08

**Authors:** Yasmeen Khafajah, Mariam Shaheen, Dania El Natour, Maxime Merheb, Rachel Matar, Jamilah Borjac

**Affiliations:** 1 Beirut Arab University, Department of Biological Sciences, Faculty of Science, Debbieh, Lebanon.; 2 Beirut Arab University, Department of Internal Medicine, Faculty of Medicine, Beirut, Lebanon.; 3 Liwa College, College of Medical and Health Sciences, United Arab Emirates.; 4 American University of Ras Al Khaimah, School of Arts and Sciences, United Arab Emirates.

**Keywords:** Parkinson Disease, Neurotransmitters, NPs, ZnO Nanoparticles, Cobalt Ferrite Nanoparticles

## Abstract

**Goals of the investigation:** This work aimed to evaluate the neuroprotective effects of zinc oxide (ZnO) nanoparticles in an experimental mouse model of rotenone-induced PD and investigate the therapeutic effects of ZnO, cobalt ferrite nanoparticles, and their combination.

**Methods:** The levels of dopamine, norepinephrine, epinephrine, and serotonin were assessed using ELISA in the control and experimental model of PD mice. The dopa-decarboxylase expression level was assayed by real-time PCR. The expression level of tyrosine hydroxylase (TH) was assessed by western blot analysis.

**Results:** Our data showed that levels of dopamine decreased in PD mice compared to normal. ZnO NP increased dopamine levels in normal and PD mice (37.5% and 29.5%; respectively, compared to untreated mice). However, ZnO NP did not cause any change in norepinephrine and epinephrine levels either in normal or in PD mice. Levels of serotonin decreased by 64.0%, and 51.1% in PD mice treated with cobalt ferrite and dual ZnO- cobalt ferrite NPs; respectively, when compared to PD untreated mice. The mRNA levels of dopa-decarboxylase increased in both normal and PD mice treated with ZnO NP. Its level decreased when using cobalt ferrite NP and the dual ZnO-cobalt ferrite NP when compared to untreated PD mice. A significant decrease in TH expression by 0.25, 0.68, and 0.62 folds was observed in normal mice treated with ZnO, cobalt ferrite, and the dual ZnO-cobalt ferrite NP as compared to normal untreated mice. In PD mice, ZnO administration caused a non-significant 0.15-fold decrease in TH levels while both cobalt ferrite and the dual ZnO-cobalt ferrite NP administration caused a significant 0.3 and 0.4-fold decrease respectively when compared to untreated PD mice.

**Principal conclusion**: This study reveals that ZnO NPs may be utilized as a potential intervention to elevate dopamine levels to aid in PD treatment.

## Introduction

Parkinson's disease (PD) is a neurodegenerative disorder characterized by cell loss in the substantia nigra which leads to disturbance in the ventral section of the pars compacta 1. PD is a prevalent and debilitating disease that affects millions of people worldwide with a significant impact on the quality of life 2. Further research into the causes and mechanisms of PD could lead to better treatments and possibly a cure for long-term neurodegenerative disease. PD is characterized by the buildup of "Lewy bodies," aberrant aggregates made up of α-synuclein protein and other unidentified filamentous proteins, within brain cells 1. The bradykinesia, rest tremors, stiffness, and postural symptoms are most distinctive with PD 3.

Neurotransmitters play an important role in transmitting alerts among neurons across synapses. Different neurotransmitters are involved in PD onset and progression including dopamine, norepinephrine, epinephrine, and serotonin 4. Catecholamine biosynthesis starts with the conversion of phenylalanine to tyrosine through the action of phenylalanine hydroxylase and then from tyrosine into biologically active compounds such as dopamine, norepinephrine, and epinephrine through a pathway that includes Tyrosine hydroxylase (TH) that catalyzes the addition of a hydroxyl group onto the benzene ring of tyrosine, yielding 3,4-dihydroxyphenylalanine (DOPA). DOPA decarboxylase (DDC) employs pyridoxal phosphate as a coenzyme to catalyze the decarboxylation of DOPA, yielding dopamine. This biogenic amine, belonging to the catecholamine family, serves as a neurotransmitter within the nervous system and affects memory, mood regulation, emotion management, and cognition 5. Dopamine undergoes hydroxylation mediated by dopamine-β-hydroxylase to yield norepinephrine (NE), also known as noradrenaline. NE has both hormonal and neuronal functions; and epinephrine, a catecholamine neurotransmitter, causes peripheral vasoconstriction and affects heartbeat in a chronotropic and inotropic manner 6. NE can be methylated to form epinephrine, commonly known as adrenaline, through the action of phenyl ethanolamine-N-methyltransferase (PNMT), utilizing S-adenosyl methionine (SAM) as a methyl donor 5. Additionally, serotonin a monoamine neurotransmitter derived from tryptophan, plays a vital role in the central nervous system, blood, and gastrointestinal tract 7. Autism 8, depression 9, and neurodegenerative diseases like PD are just a few complicated behavioral syndromes that have been linked to changes in serotonin transmission.

Rotenone is a naturally occurring toxin 10, and in animal models, it has been utilized to mimic PD-like symptoms such as α-synuclein accumulations 11, prolonged microglial activation 12 and microglial phagocytosis 13, and inhibition of proteasomal function 14. Rotenone has also been shown to induce PD at high dosages by inflicting long-lasting damage on the nigrostriatal pathway 15. Rotenone produces developed behavioral traits such as bent posture, hypokinesia, and crucial stiffness 16. Rotenone inhibits complex I of the mitochondrial respiratory chain (MRC) by suppressing electron transport from complex I's iron-sulfur centers to ubiquinone resulting in a blockage of oxidative phosphorylation and reduced ATP generation. Furthermore, the inadequate electron transport to oxygen results in the creation of reactive oxygen species (ROS) that cause damage to the mitochondria eventually leading to apoptosis 17,18.

Since there is currently no treatment for PD, existing drugs are designed to treat its symptoms, there is rising interest in the use of nanoscience and nanotechnology for the prevention and treatment of neurological illnesses including PD 19. Nanoparticles (NP) like zinc oxide (ZnO) 20 and cobalt ferrite (CoFe_2_O_4_) 21 show promise in a variety of applications, including the delivery of drugs and the identification of biomarkers. These NPs can enter the brain, especially the cerebellum, cortex, and olfactory bulb, which increases their medication bioavailability 22,23. In this study, our objective is to evaluate the effects of experimental interventions utilizing ZnO and CoFe_2_O_4_ nanoparticles on the levels of various neurotransmitters in a mouse model of rotenone-induced Parkinson's disease.

## Methods

### Animals

Healthy male Balb/c mice weighing 20-25 g were obtained from the Beirut Arab University animal facility. The mice were housed under standard conditions at 22 ± 2°C under a 12 h light/dark cycle with free access to a standard pellet diet and water. The mice were left to acclimate for one week before beginning the experiments. Experimental procedures on animals were approved and carried out following the guidelines proposed by the Institutional Review Board (IRB) at Beirut Arab University.

### Experimental Design

Animals were divided into eight groups of 6 mice each (n-6/group) as shown in Table 1. Establishing Parkinsonian behavior in mice was performed according to the protocol published by Cannon *et al.* 24. Rotenone (Sigma-Aldrich Co, USA, R.8875) was suspended in saline. Mice were subcutaneously injected with 1.5 mg/kg of rotenone every other day for 12 days 25. ZnO NP and CoFe_2_O_4_ NP were prepared by the physics department at Beirut Arab University according to the protocol published by Shahbaz *et al.* 26. NPs were suspended in saline at a final concentration of 1.6 mg/ml and injected intraperitoneally. Normal or PD mice were given 8 doses of NPs at a concentration of 5.6 mg/kg every other day for 16 days. The chosen dosage was based on a previous study 27. Table 1 summarizes the effects of experimental interventions taken by each group. The weight of mice was recorded at the beginning of the experiment and over the whole period of the study. Two days following the last NP application, mice were sacrificed and their brains were collected, washed with ice-cold saline (0.9%), weighed, and stored at -80 °C for later analyses.

### Tissue Homogenization

Whole brains were homogenized in Sucrose Hepes Tris-Base buffer (250mM sucrose-10mM Hepes-50mM Tris-Base buffer, pH 7.4) supplemented with a protease inhibitor cocktail at a ratio of 3 ml of buffer per 1 mg of tissue. The homogenate was then centrifuged for 10 min at 3000 rpm at 4^o^C. The supernatant was stored at - 80 °C for later use.

### Protein Quantification

Protein quantification was performed using Lowry assay according to the manufacturer's protocol. In brief, 50µL of samples were mixed with 1000 µL of Lowry reagent (Solution containing 1% CuSO_4_, 2% sodium potassium tartrate prepared in 0.1 M NaOH) and then incubated for 15 minutes at room temperature. 100µL of diluted Folin reagent was then added and the mixture was re-incubated for an additional 30 min at room temperature in the dark before measuring the absorbance of the developed color at 650 nm. Concentrations were determined from a standard curve obtained using different dilutions of bovine serum albumin (BSA, 0.02%). The assay was performed in triplicates and the average of the three readings was used to plot the standard graph to determine protein concentration.

### Quantification of TH expression by Western Blot Analysis

Protein expression was quantified using enhanced chemiluminescence (ECL kit), and protein bands were assessed using NIH Image J software. They were first standardized to their respective GAPDH band, and then fold expression was determined relative to the control. Anti-GAPDH antibody [GT239] (HRP) ab184193, Anti-Tyrosine Hydroxylase (TH) antibody ab51191, and Goat Anti-Rabbit IgG H&L (HRP) ab6721 were purchased from Abcam^®^. Primary antibodies were diluted according to the manufacturer (1:1000). Secondary antibodies were diluted 1:10000 in the blocking buffer.

### Quantification of DDC expression by RT-PCR

The RNeasy Plus Mini Kit was used to extract total RNA from brain homogenates according to manufacturer recommendations. Preparation of cDNA was done using QuantiTect Reverse Transcription Kit (QIAGEN®) which was used to reverse-transcribe 1 ug of the purified RNA. The expression of DDC was quantified by RT-PCR using QuantiFast SYBR Green PCR Kit (QIAGEN®). Gene expression was measured by comparative threshold cycle (Ct) methods using glyceraldehyde-3 phosphate dehydrogenase (GAPDH) as a reference gene. During each assay, the mean Ct (mCt) values were determined. DCt value was determined as the difference between the Ct of DDC and the Ct of GAPDH genes. The relative quantity of DDC gene expression compared to the GAPDH gene was calculated applying the gene dosage ratio formula (GDR = 2^-DDCt^) where: DDCt = (mCt DDC - mCt GAPDH) normal sample - (mCt DDC - mCt GAPDH) test sample. Gel electrophoresis was used to visualize the amplified PCR products and primer specificity. The primers used to amplify the DDC gene (F: 5'TCACCAAGGAGAGAGAGAGAGC3', R: 5'GACCACAAAGAATGGAATCAGG3') and GAPDH F: 5'TCACCAAGGAGAGAGAGAGAGC3', R: 5'GACCACAAAGAATGGAATCAGG3') were synthesized by TIB MOLBIOL, Berlin, Germany.

### RNA Extraction

Total RNA was extracted from the brains of all studied groups. The quality of the extracted RNA was assayed by electrophoresis using 1% agarose gel. RNA concentration was determined by measuring its absorbance at 260 nm.

### Quantification of Neurotransmitters

The levels of epinephrine, norepinephrine, and dopamine were determined using 3-CAT Research Enzyme Immunoassay kits (Labor Diagnostika Nord (LDN)^ ®^) used according to the manufacturer's instructions. The determination of serotonin levels was performed using the Serotonin Research ELISA kit (Labor Diagnostika Nord (LDN)^ ®^) also following the manufacturer's instructions. Quantification of unknown samples was done by comparing their absorbance with a standard curve prepared using known standard concentrations of the different neurotransmitters.

### Statistical Analysis

All statistical analyses were performed with the standard statistical program Graph Pad Prism Ver. 9. The data are expressed as mean ± standard deviation. In all tests, the minimum criterion for statistical significance was p < 0.05. Statistical analysis of the obtained results was analyzed using (ANOVA). p-values of less than 0.05 were considered significant. The results with **** indicate the significance at p<0.0001, *** at p<0.001, ** at p<0.01and * at p<0.05.

## Results

### Induction of PD

**Figure 1:**
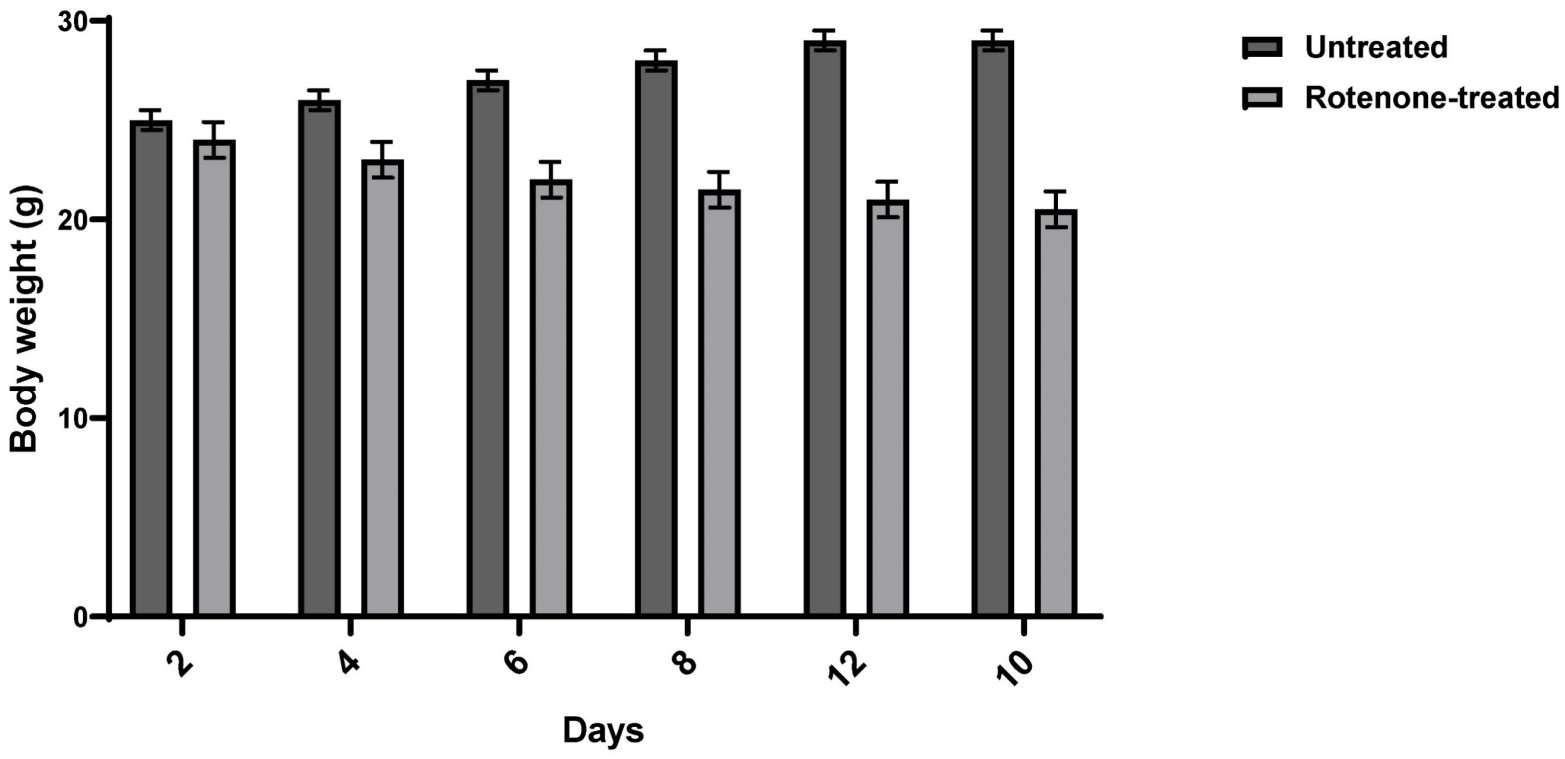
** Weight Change after Rotenone Administration.** Balb/c mice were injected with rotenone at 1.5 mg/kg. Data shows an average weight of 6 mice per group ± SD.

PD was induced in three-week-old Balb/c male mice using rotenone. Mice were injected subcutaneously with rotenone at 1.5 mg/Kg every other day for 12 days. The appearance of PD symptoms in mice was assessed by monitoring their postural instability, bradykinesia, gait, rigidity, and weight changes. All rotenone-treated animals exhibited Parkinsonian symptoms. Rotenone administration caused a significant 18.5% weight loss compared to normal mice. **Figure 1** illustrates the weight changes following rotenone administration.

### TH expression levels

**Figure 2:**
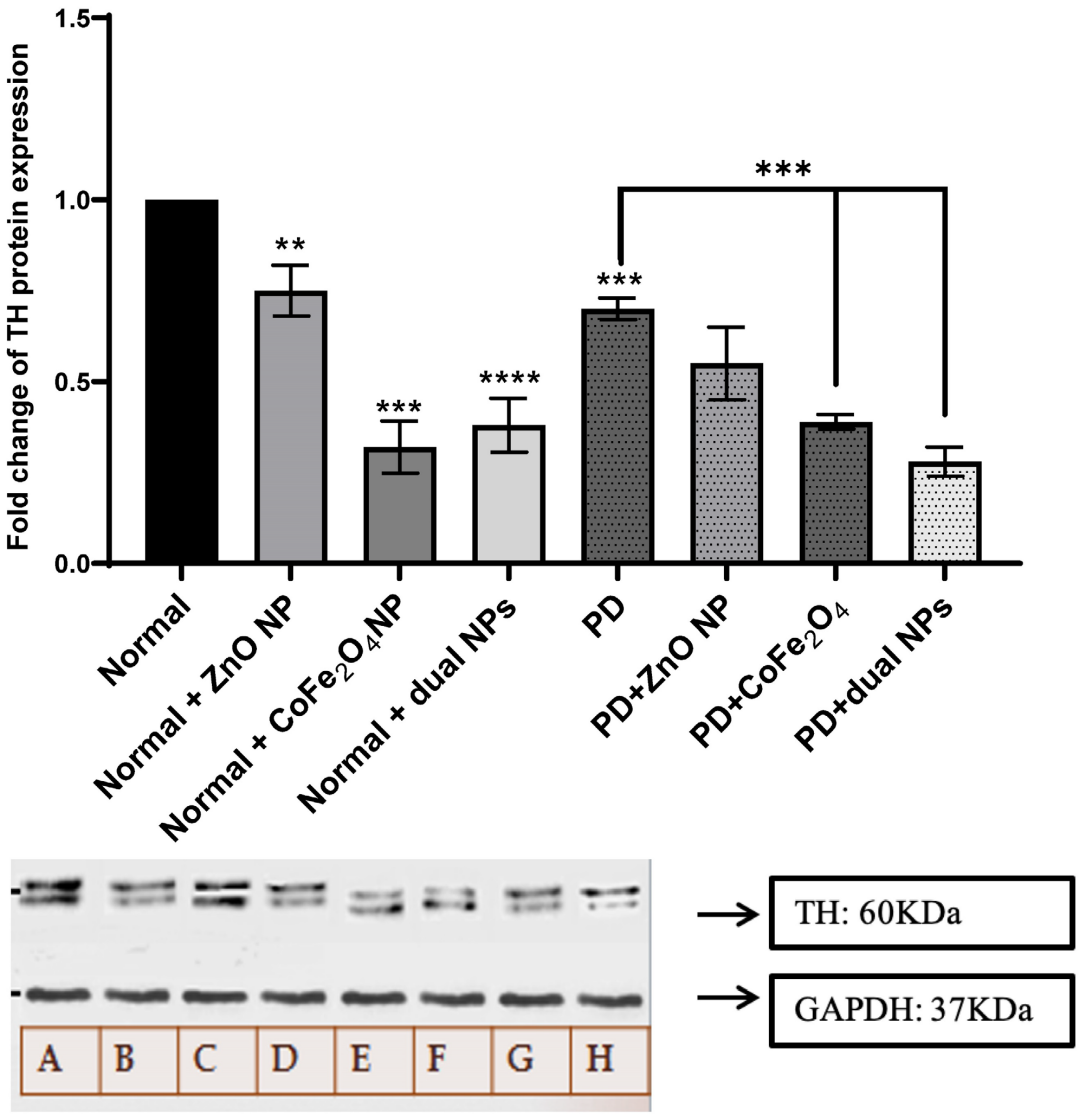
** Effect of NP on the expression of tyrosine hydroxylase**. Normal and PD mice were treated with ZnO, CoFe_2_O_4_, or their combination. TH expression was assayed using western blot analysis. A quantitative assessment of bands was done using ImageJ. Expression levels of treated samples and controls were normalized to their respective GAPDH, and then fold expression was determined relative to the control. A: Normal, B: Normal+ ZnO NP, Normal+ CoFe_2_O_4_ NP, D: Normal+ dual NP, E: PD, F: PD+ ZnO NP, F: PD+CoFe_2_O_4_ NP, PD+ dual NP. The data presented is the mean ± SD of determinations from different experiments. Asterisks on bars represent significance relative to the control, and those drawn upwards represent inter-categorical statistical significance. (*), (**), (***), and (****) correspond to P < 0.05, 0.01, and 0.001; respectively.

TH catalyzes the hydroxylation of tyrosine to L-DOPA. It catalyzes the rate-limiting step in dopamine, epinephrine, and NE synthesis. Variation in its expression will affect the rate of catecholamine synthesis. Figure 2 shows the variation in TH expression in normal and PD mice with and without NP administration. TH level decreased in PD mice by 0.3 folds compared to normal mice (p-value 0.0003). Similarly, a decrease was observed in normal mice treated with the ZnO NP, CoFe_2_O_4_ NP, and the combination of both NPs by 0.25, 0.68, and 0.62 folds respectively (p-values 0.0078, 0.0001, and <0.0001). Furthermore, TH level in PD mice treated with ZnO NP showed a non-significant decrease by 0.15-fold (p-value 0.1103), a significant decrease with CoFe_2_O_4_ NP, and the dual NP administration by 0.311-fold (p-value 0.0004) and 0.42 (p-value 0.0003) respectively compared to PD mice, as shown in **Figure 2.** This shows that these NPs have inhibitory effects on the expression of TH.

### DDC expression levels

**Figure 3:**
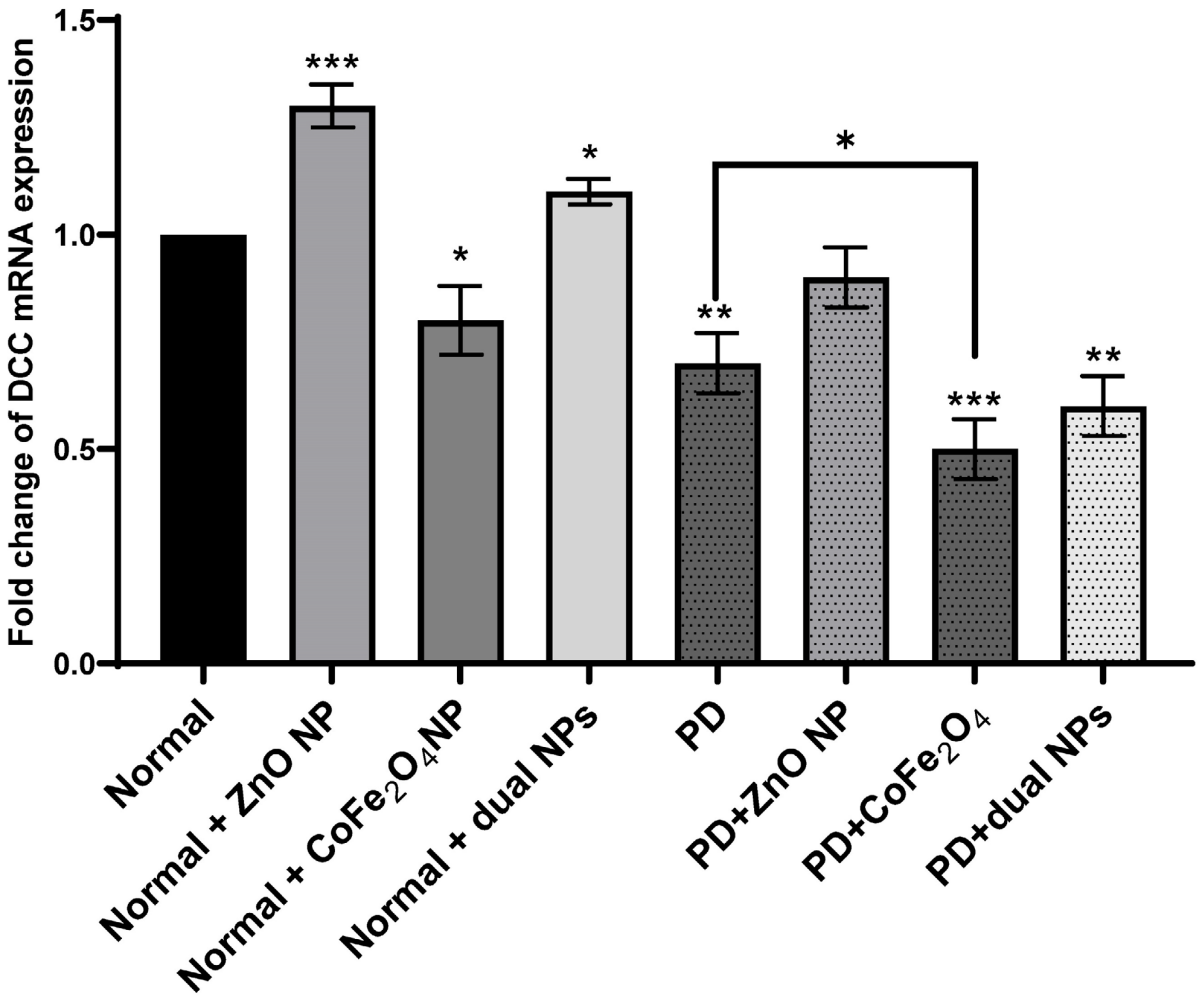
** Effect of NP on the expression of DDC.** Normal and PD mice were treated with ZnO, CoFe_2_O_4_, or their combination. DDC mRNA expression was detected by RT-PCR. The housekeeping gene GAPDH served as a control to calibrate the cDNA template for all the samples. Relative quantification analysis was performed using the comparative Ct method (2^-ΔΔCT^). Each value is shown as a mean of three determinations ± SD. Asterisks on the bars represent significance relative to the control (*), (**), and (***) correspond to p< 0.05, *p* < 0.01, and *p* < 0.001 respectively.

Evaluation of relative DDC gene expression between normal and PD mice with and without NP administration was determined through the difference in their Ct values during the exponential phase of amplification. mRNA levels of DDC in normal mice treated with ZnO NP increased 0.3-fold (p-value 0.0009) and 0.1-fold when treated with dual NPs (p-value 0.018) as compared to normal untreated mice. However, the DDC expression level decreased 0.2-fold (p-value 0.024) when mice were treated with CoFe_2_O_4_ NP alone. Expression of the DDC gene in PD mice decreased by 0.3-fold (p-value 0.003) compared to normal mice. Furthermore, DDC expression decreased in PD mice compared to normal mice treated with ZnO, CoFe_2_O_4_, and dual administration by 0.1, 0.5, and 0.4 folds (p-values 0.15, 0.003, 0.011) respectively. However, upon comparing NP-treated PD mice to untreated PD mice, it is noticed that DDC mRNA levels increased by 0.2-fold (p-value 0.05) with ZnO NP. However, it decreased by 0.2-fold (p-value 0.032) and 0.1-fold (p-value 0.2.) with CoFe_2_O_4_ and dual NP administration, respectively, proving that ZnO NPs may have an activating effect on DDC expression versus an inhibitory effect by CoFe_2_O_4_ NP (**Figure 3**).

### Dopamine Level

**Figure 4:**
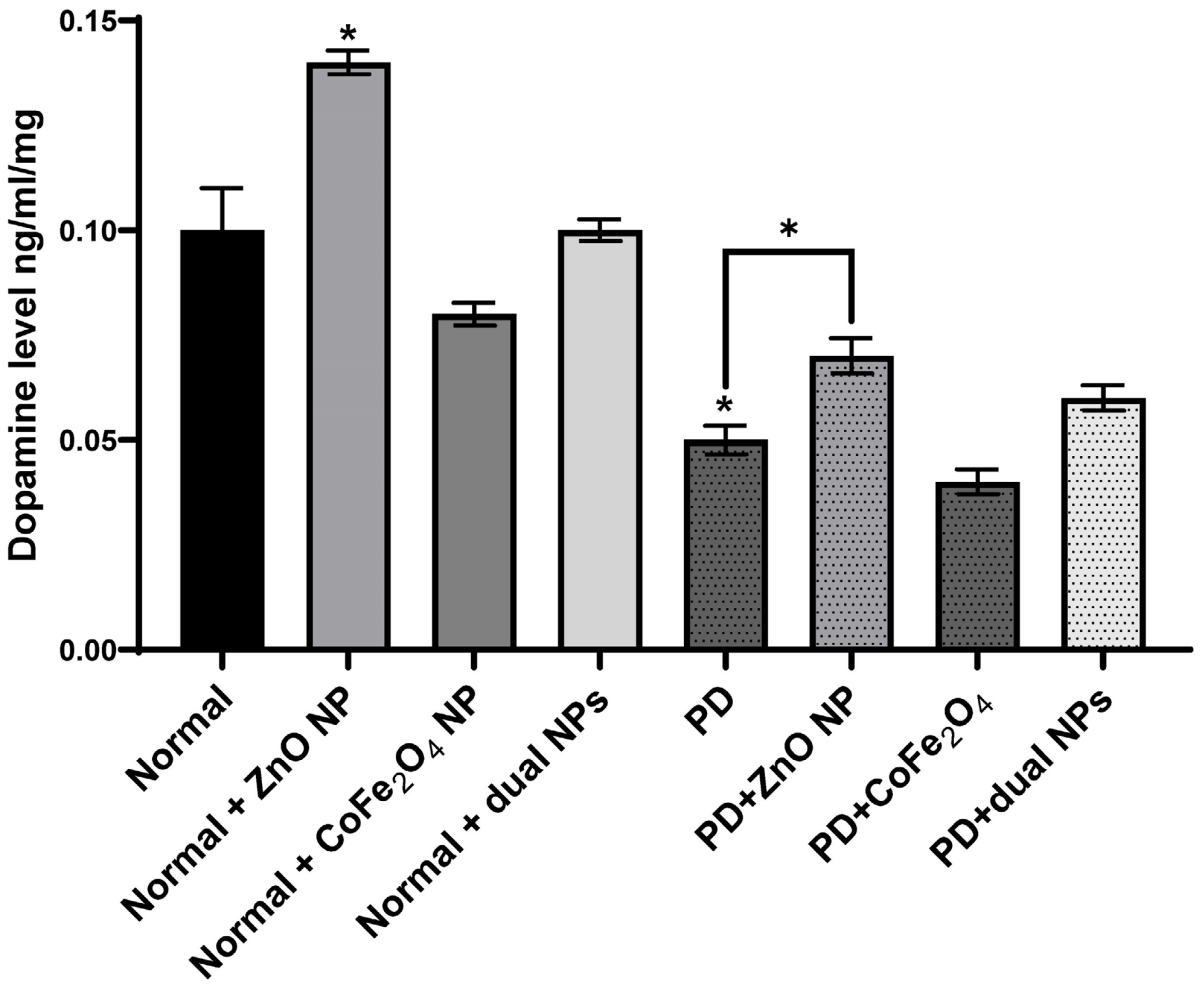
**Effect of rotenone and NP on dopamine level in the brain of Balb/c mice.** Normal and PD mice were injected with 5.6 mg/kg ZnO, 5.6 mg/kg CoFe_2_O_4_, or a combination of both NPs. Levels of dopamine were assayed in whole brain extracts using the ELISA method. Data represented are the average of 3 different determinations ±SD. Asterisks on bars represent significance relative to the control and those drawn-up wards represent intra-categorical statistical significance. (*) corresponds to *p* < 0.05.

Dopamine levels were measured in the brains of normal and rotenone-induced PD mice with and without NP administration. The level of dopamine in the brains of PD mice decreased significantly compared to normal mice (53.81% ± 0.0034, p-value 0.0156). The level of dopamine in normal mice injected with ZnO NP (at 5.6mg/Kg) increased significantly compared to normal mice (37.52% ± 0.0028 increase, p-value 0.0474). However, the levels of dopamine in normal mice injected with CoFe_2_O_4_ NP (at 5.6mg/Kg) or a combination of both NPs decreased by 15.1% ± 0.0027 and 3.39% ± 0.0056 respectively. This decrease was not significant either for CoFe_2_O_4_ NP (p-value 0.3186) or for the combination of both ZnO and CoFe_2_O_4_ NP (p-value 0.8147). However, upon treating PD mice with ZnO NP or a combination of both NPs, the level of dopamine increased compared to untreated PD mice. Similarly, a significant increase was observed when treated with ZnO NP (29.49% ±0.0042, p-value 0.0104), while it was not significant when treated with the combination of both NPs (11.66%, ± 0.00302, p-value 0.10). On the other hand, administration of CoFe_2_O_4_ NP to PD mice resulted in a non-significant alteration in dopamine levels compared to PD mice that did not receive the nanoparticles (**Figure 4**).

### Norepinephrine Level

**Figure 5:**
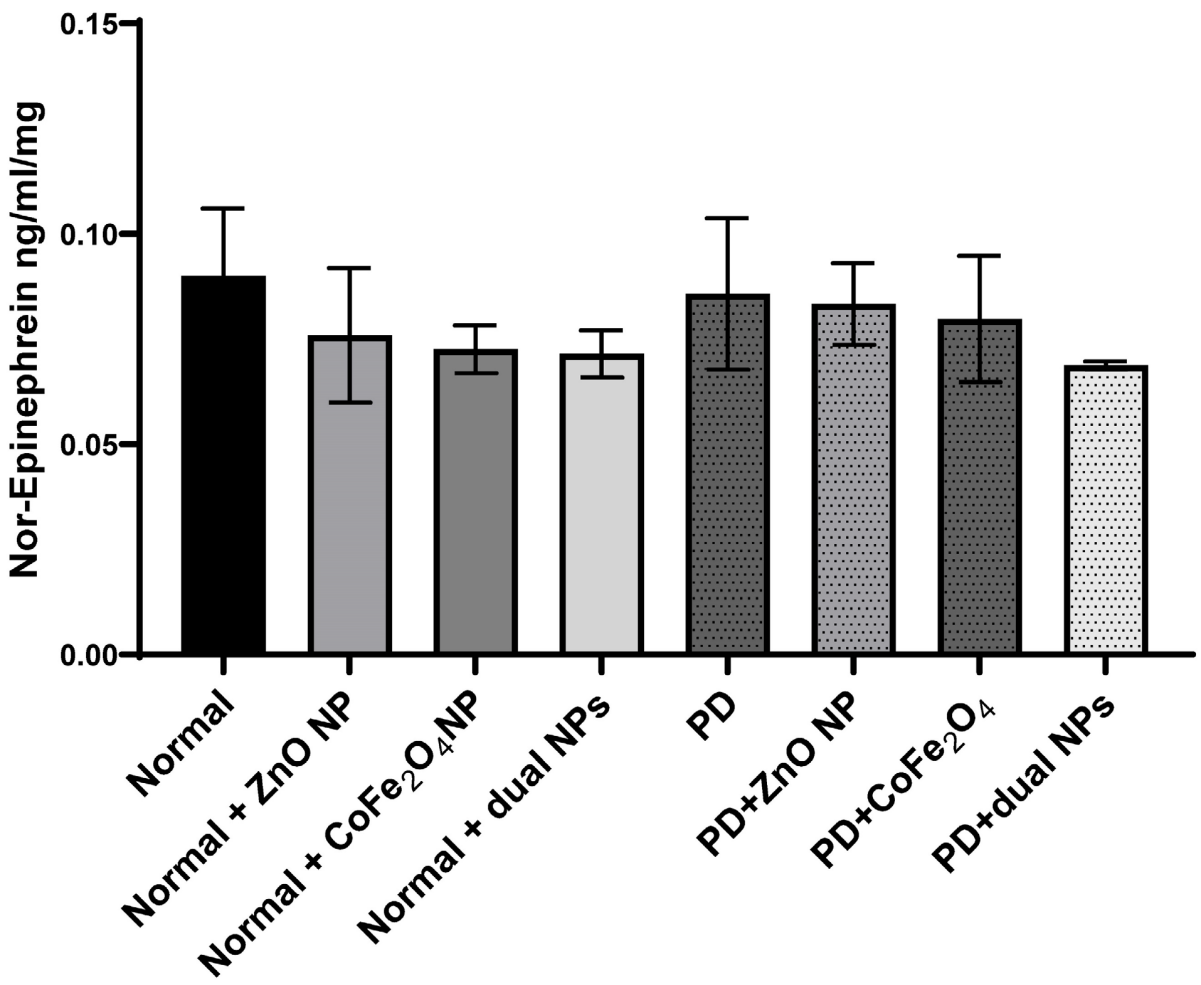
** Effect of ZnO NPs, CoFe_2_O_4_ NPs, and their combination on the levels of Norepinephrine in the brain of Balb/c mice.** Normal and PD mice were injected with 5.6 mg/kg ZnO, 5.6 mg/kg CoFe_2_O_4_, or a combination of both NPs**.** Levels of Norepinephrine were assayed in whole brain extracts using the ELISA method. Data represented are the average of 3 different determinations ±SD.

Levels of norepinephrine were measured in the brains of all studied groups. Norepinephrine levels in PD mice showed no significant reduction by 4.7% ± 0.018 (p-value 0.808) compared to normal mice. Similarly, the level of norepinephrine in normal mice injected with ZnO NP, or CoFe_2_O_4_ NP, or the combination of both NPs showed no significant reduction by 15.1% ± 0.016 (p-value 0.43), 19.4% ± 0.057 (p-value 0.257), and 20.6% ± 0.0056 (p-value 0.232) respectively as compared to normal mice. Treating PD mice with ZnO NP, CoFe_2_O_4_ NP or their combinations also leads to a non-significant decrease in the norepinephrine levels. The level of norepinephrine decreased by 7.44% ± 0.0097 (p-value 0.605), 11.44% ± 0.015 (p-value 0.488), and 23.66% ± 0.001 (p-value 0.114) for ZnO NP, CoFe_2_O_4_ NP, and the dual NP administration respectively (**Figure 5**).

### Epinephrine Level

**Figure 6:**
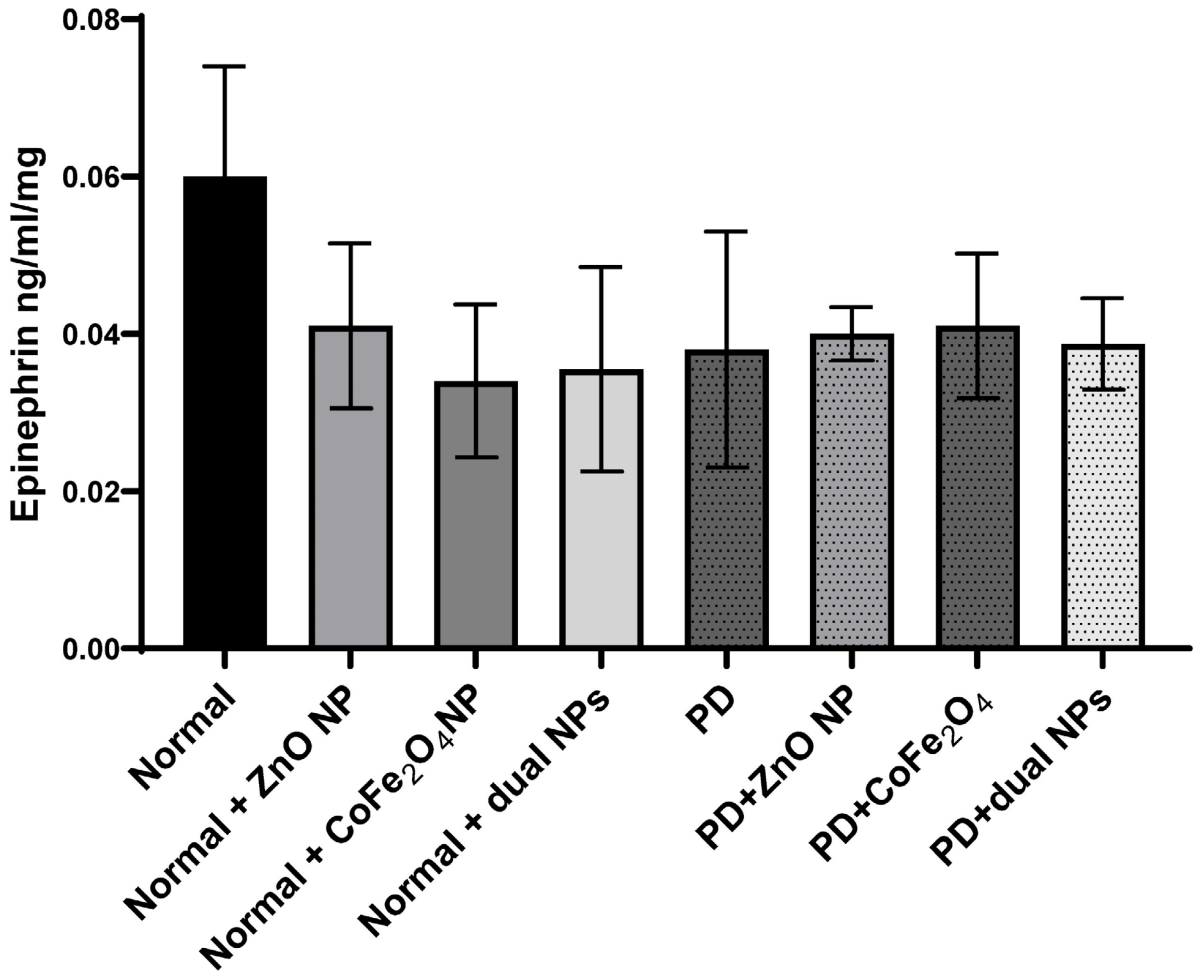
** Effect of ZnO NPs, CoFe_2_O_4_ NPs, and their combination on the levels of Epinephrine in the brain of Balb/c mice.** Normal and PD mice were injected with 5.6 mg/kg ZnO, 5.6 mg/kg CoFe_2_O_4_, or a combination of both NPs. Levels of epinephrine were assayed in whole brain extracts using the ELISA method. Data represented are the average of 3 different determinations ±SD.

Similarly, levels of epinephrine were also measured in the brains of the different animal groups. Epinephrine levels decreased by 36.03% ±0.015 in PD mice compared to normal mice. However, this decrease was not significant (p-value 0.2862). The level of epinephrine in normal mice injected with ZnO NP, CoFe_2_O_4_ NP, or a combination of both NPs decreased by 30.9% ± 0.0105 (p-value 0.32), 43.9% ± 0.0097 (p-value 0.18), and 40.7% ±0.013 (p-value 0.22); respectively, compared to control mice. However, upon treating PD mice with ZnO NP (group PD + ZnO NP), CoFe_2_O_4_ NP (group PD+ CoFe_2_O_4_ NP), and dual NPs (group PD+ dual NPs), a nonsignificant increase in epinephrine levels was observed. ZnO administration caused a 1.7% ±0.0034 (p-value 0.944) increase, CoFe_2_O_4_ led to an increase of 9.3% ± 0.0092 (p-value 0.74), and the dual NP administration caused a 2.1 % ± 0.0058 (p-value 0.93) increase if compared to the PD group as shown in** Figure 6.**

When norepinephrine and epinephrine were compared in PD mice to normal mice, both showed a non-significant decrease. In normal mice receiving nanoparticles, both norepinephrine and epinephrine levels reduced dramatically as compared to control animals. However, the fall in epinephrine levels was often greater than that in norepinephrine, indicating a possible differential in their responses to nanoparticle exposure. There were no significant differences in norepinephrine and epinephrine levels after nanoparticle delivery compared to the PD group. Notably, there were small increases in epinephrine levels following nanoparticle delivery, although norepinephrine levels remained largely steady.

### Serotonin Level

**Figure 7:**
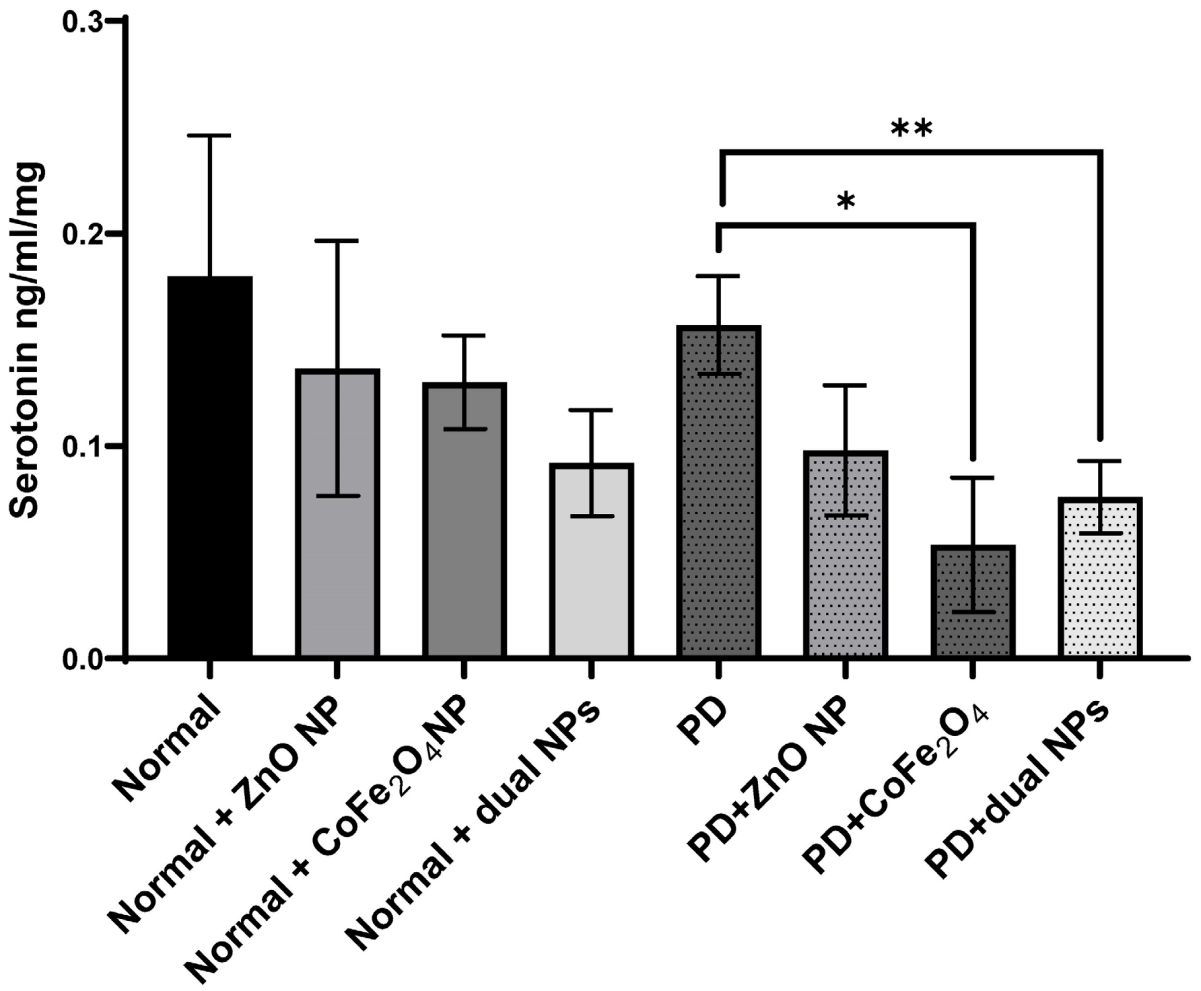
** Effect of ZnO NPs, CoFe_2_O_4_ NPs, and their combination on the levels of Serotonin in the brain of Balb/c mice.** Normal and PD mice were injected with 5.6 mg/kg ZnO, 5.6 mg/kg CoFe_2_O_4_, or a combination of both NPs. Levels of serotonin were assayed in whole brain extracts using the ELISA method. Data represented are the average of 3 different determinations ±SD. Asterisks on bars represent significance relative to the control and those drawn-up wards represent intra-categorical statistical significance. (*) and (**) correspond to p < 0.05, 0.01 respectively.

Serotonin levels were measured in the brains of all mice in the 8 groups. The level of serotonin in rotenone-treated mice showed no significant reduction by 12.8% ± 0.023 (p-value 0.597) compared to normal mice. Similarly, the level of serotonin in normal mice treated with ZnO NP, CoFe_2_O_4_ NP, or the combination of both NPs showed no significant reduction by 24.06% ± 0.06, 27.77% ±0.022, and 48.45% ± 0.025 with p-values of 0.445, 0.281, and 0.099; respectively, as compared to control mice. Levels of serotonin in PD mice treated with ZnO NP, CoFe_2_O_4_ NP, or the dual NPs exhibited a decrease of 37.1% ± 0.0305, 64.0% ± 0.0316, and 51.1% ± 0.017; respectively, compared to PD mice. The decrease due to ZnO NP administration was not significant (p-values 0.056), while the decrease due to CoFe_2_O_4_ NP or the dual NP administration was significant, with p-values of 0.01, and 0.008 respectively **(Figure 7).**

## Discussion and Conclusion

PD is a prevalent neurodegenerative disorder that is characterized by cell loss in the substantia nigra mainly disturbing the ventral section of the pars compacta. Attempts to find a cure for PD are still under investigation till now and different animal models and chemicals have been used to mimic PD symptoms including rotenone. Rotenone inhibits complex I of the mitochondrial respiratory chain and suppresses the electron transport from complex I's iron-sulfur centers to ubiquinone, which results in a blockage of oxidative phosphorylation, and reduced ATP generation which can cause mitochondrial damage and apoptosis 17,18. Currently, the drugs used aimed at increasing the levels of dopamine or inhibiting this breakdown 28, such as Levodopa can cross the blood-brain barrier (BBB) and metabolize to dopamine by the action of DDC 29 or AtreMorine, a recent natural drug used to treat PD patients that display a novel neuroprotective agent for dopaminergic neurons with potential prophylactic and therapeutic activity in PD 30. Technologies based on NPs have evolved as platforms for delivering and dispensing therapeutic payloads to the target location. By carefully selecting the composition and physicochemical characteristics that guarantee NP's biological behavior, good cellular uptake, and drug delivery capability while avoiding toxicity, this goal may be achieved 31. Recently the use of NPs has been implemented to treat a diversity of different neurological disorders. Due to their size, selectivity, and their ability to cross BBB; NPs may be considered as ideal therapeutic targets in neurodegenerative disorders treatment 19.

This study aims to investigate whether ZnO NPs, CoFe_2_O_4_ NPs, or their combination have any impact on the level of neurotransmitters in the experimental mouse model rotenone-induced PD. Xie *et al.* proposed in their study that ZnO NPs may perform several functions in the CNS of mental diseases 32 and zinc acts as a pro-antioxidant mediator and exerts antioxidant activities in different tissues including the brain, and is known to be a cofactor for different metabolic enzymes 33. CoFe_2_O_4_ NP exhibits diverse applications in medicine, food preservation, electrochemistry, and water treatment, being utilized for antimicrobial activity, cancer therapy, wound healing, and drug delivery in the medical field 34 but their impact remains unexplored in neurological disorders. Notably, cobalt, a component of CoFe_2_O_4_, is recognized as a neurotoxin 35 associated with neurological impairments 36 and cardiac diseases 37. Thus, we aimed to investigate the molecular mechanisms and potential therapeutic applications of CoFe_2_O_4_ NPs in PD.

Catecholamines, including dopamine, norepinephrine, and epinephrine, are produced from tyrosine by a sequence of enzymatic processes in the nerve system's chromaffin cells and adrenal medulla. TH converts tyrosine to L-DOPA, which is then decarboxylated by DDC to form dopamine 38. Dopamine is then hydroxylated to yield norepinephrine, which is finally methylated to produce epinephrine, with each step requiring specific cofactors and regulatory mechanisms. Starting with TH, which catalyzes the rate-limiting step in catecholamine synthesis, our results show that ZnO and CoFe_2_O_4_ NP administration of PD mice had no significant change in TH expression level compared to PD mice. However, the dual NP administration exhibited a significant decrease in TH expression level. This means that the administration of dual NPs can affect negatively dopamine synthesis as confirmed by dopamine measurement (Figure 4). ZnO NPs did not exert an effect on the expression of this enzyme showing that it does not cause a decrease in dopamine level as confirmed by Amara *et al.* 39. DDC is not the rate-limiting step in dopamine synthesis. However, it becomes the main control step in PD patients treated with L-DOPA. The results show that DDC mRNA expression increases in PD mice treated with ZnO NP driving the pathway to more dopamine synthesis as confirmed by dopamine measurement. Our results have shown that the level of dopamine in PD mice increased significantly with ZnO NP administration implying its possible use to treat PD. On the other hand, the use of CoFe_2_O_4_ alone or in combination with ZnO affected dopamine levels. As for the effect of both NPs and their combination on the levels of epinephrine norepinephrine and serotonin, no significant changes were observed. Both norepinephrine and epinephrine levels showed some changes in PD mice exposed to nanoparticles, there are differences in the magnitude and significance of these changes, suggesting potential variations in their regulatory pathways and responses to external stimuli such as nanoparticle administration. But still, it is expected that these two neurotransmitters increase concomitantly with dopamine as it is their precursor.

It might be possible that these NPs inhibit dopamine hydroxylase leading to a decreased level of both norepinephrine and epinephrine. Furthermore, our results show that the level of serotonin did not change significantly in PD mice treated with Zn compared to PD mice. However, CoFe_2_O_4_ NPs and dual NP administration in PD mice lead to a significant decrease in serotonin compared to control PD mice. This result might be expected as cobalt can mediate the depletion of neurotransmitters such as dopamine, noradrenaline, and serotonin and can cause presynaptic blockage of calcium channels 40.

Our results are consistent with earlier research in the area. A recent, for instance, showed that ZnO NPs exerted neuroprotective actions in spinal cord injury. Following the administration of ZnO NPs, the researchers saw a reduction in oxidative stress levels and rescuing the neuronal apoptosis 41 aligning with another study in 2022, that showed ZnO NP possessed neuroprotective action by reducing the basal levels of reactive oxygen species, *Bax/Bcl-2* mRNA ratios, p53 and apoptosis brought by 6-OHDA in SH-SY 5Y cells (neuroblastoma cell line)^42^. Another study in 2012, explored the neuroprotective effects of ZnO NP on spatial cognition and synaptic plasticity in mice with depressive-like behaviors 32. It is important to keep in mind that most of the research has shown conflicting findings to ours as most of the results showed the ZnO NP exerts neurotoxic effects 23,43-45. Initially, it's important to mention that variations in the experimental results among the studies may be owed to the treatment protocols nanoparticle size and dose used, as smaller sizes of ZnO nanomaterials showed higher toxicity in treating PD-like symptoms 43.

Future research ought to focus on unraveling the underlying processes by which ZnO and cobalt ferrite nanoparticles exert their neuroprotective effects to advance our knowledge of the therapeutic potential of nanoparticles in PD. Additionally, comparative research comparing the effectiveness of various nanoparticles in comparable experimental models would shed light on their respective merits and drawbacks. Although the current study shows that ZnO nanoparticles have neuroprotective properties and have the potential to raise dopamine levels in PD, it is important to take into account the whole body of research in this field. We may get a more thorough knowledge of the therapeutic potential of nanoparticles and pinpoint areas for additional research and improvement of nanoparticle-based therapies in PD by analyzing and contrasting the results from multiple studies. This study has certain limitations, despite its encouraging results, which should be noted. First, our study was done on animal models, although they provide valuable insights into the molecular mechanisms, they may not fully replicate the complexity and progression of PD in humans and the findings can't be broadly generalized. Because of the study's emphasis on animal models, further investigations are required to determine the treatment's safety and effectiveness in humans. Furthermore, our study didn't include any behavioral or histological analyses, both of which are crucial for assessing PD onset and progression and evaluating the effectiveness of the experimental interventions. Dopamine, epinephrine, NE, and serotonin levels were the main subjects of our investigation. PD, on the other hand, involves a variety of pathogenic processes, including inflammation, oxidative stress, and mitochondrial dysfunction. To get a more thorough grasp of their therapeutic potential, future research should investigate the wider influence of NP therapies on these factors. The short research duration may have led to missing the potential long-term impacts of NP therapies. Long-term studies are essential to PD since it is a chronic disorder that worsens with time. These limitations highlight the necessity of more investigation to fill up these knowledge gaps and provide a thorough grasp of the therapeutic potential of NPs for PD.

## Data Availability

The datasets utilized or analyzed in the present study can be obtained from the corresponding author upon reasonable request.

## Figures and Tables

**Table 1:** Description of experimental interventions taken in each study group

Group	Description
1	Normal mice injected with saline the vehicle used to suspend the NPs and served as control.
2	Mice were injected with rotenone to induce PD and then injected with saline which served as control.
3	Normal mice were treated with 8 doses of ZnO NP at 5.6 mg/kg for 16 days.
4	PD mice were treated with 8 doses of ZnO NP at 5.6 mg/kg for 16 days.
5	Normal mice were treated with 8 doses of CoFe_2_O_4_ NP at 5.6 mg/kg for 16 days.
6	PD mice were treated with 8 doses of CoFe_2_O_4_ NP at 5.6 mg/kg for 16 days.
7	Normal mice were treated with both ZnO and CoFe_2_O_4_ NP at 5.6 mg/kg for 16 days.
8	PD mice were treated with both ZnO and CoFe_2_O_4_ NP at 5.6 mg/kg for 16 days.

## Abbreviations

BBB: Blood Brain Barrier
CNS: Central Nervous System
CoFe_2_O_4_: Cobalt Ferrite
DDC: Dopa Decarboxylase
GAPDH: Glyceraldehyde-3Phosphate Dehydrogenase
NE: Noradrenaline
NP: Nanoparticles
PD: Parkinson's disease
ROS: Reactive Oxygen Species
TH: Tyrosine Hydroxylase
ZnO: Zinc Oxide
